# Composition, Structure, and Digestive Dynamics of Milk From Different Species—A Review

**DOI:** 10.3389/fnut.2020.577759

**Published:** 2020-10-06

**Authors:** Debashree Roy, Aiqian Ye, Paul J. Moughan, Harjinder Singh

**Affiliations:** Riddet Institute, Massey University, Palmerston North, New Zealand

**Keywords:** milk, composition, digestion, curd, protein, fat, structure, stomach

## Abstract

**Background:** The traditional dairy-cattle-based industry is becoming increasingly diversified with milk and milk products from non-cattle dairy species. The interest in non-cattle milks has increased because there have been several anecdotal reports about the nutritional benefits of these milks and reports both of individuals tolerating and digesting some non-cattle milks better than cattle milk and of certain characteristics that non-cattle milks are thought to share in common with human milk. Thus, non-cattle milks are considered to have potential applications in infant, children, and elderly nutrition for the development of specialized products with better nutritional profiles. However, there is very little scientific information and understanding about the digestion behavior of non-cattle milks.

**Scope and Approach:** The general properties of some non-cattle milks, in comparison with human and cattle milks, particularly focusing on their protein profile, fat composition, hypoallergenic potential, and digestibility, are reviewed. The coagulation behaviors of different milks in the stomach and their impact on the rates of protein and fat digestion are reviewed in detail.

**Key findings and Conclusions:** Milk from different species vary in composition, structure, and physicochemical properties. This may be a key factor in their different digestion behaviors. The curds formed in the stomach during the gastric digestion of some non-cattle milks are considered to be relatively softer than those formed from cattle milk, which is thought to contribute to the degree to which non-cattle milks can be easily digested or tolerated. The rates of protein and fat delivery to the small intestine are likely to be a function of the macro- and micro-structure of the curd formed in the stomach, which in turn is affected by factors such as casein composition, fat globule and casein micelle size distribution, and protein-to-fat ratio. However, as no information on the coagulation behavior of non-cattle milks in the human stomach is available, in-depth scientific studies are needed in order to understand the impact of compositional and structural differences on the digestive dynamics of milk from different species.

## Introduction

Milk has evolved to meet the nutritional and physiological requirements of the neonate. Milk is thus regarded as a high-quality food, nutritionally. Humans are known to have consumed cattle (*Bos taurus*, cow) and non-cattle (such as goat and sheep) milks as part of their diet since prehistoric times (1, 2). As a convenient source of nutrition, cattle milk is the most-consumed milk worldwide because of its widespread availability and large production volumes. Non-cattle milks are of nutritional importance to people in developing countries as well as in geographical areas in which the natural climate is unsuitable for the survival of dairy cattle (3, 4). For example, buffalo milk in Asia, sheep milk in Europe and the Mediterranean basin (including the Middle East), camel milk (“the white gold of the desert”) in Africa, goat milk (“the cattle of the poor”) in Africa and southern Asia, horse milk in the steppe areas of central Asia, yak milk on the Tibetan plateau, reindeer milk in northern Scandinavia, musk ox milk in the Arctic, and mithun milk in the hilly regions of the Indian subcontinent (3, 5).

**Figure 1:**
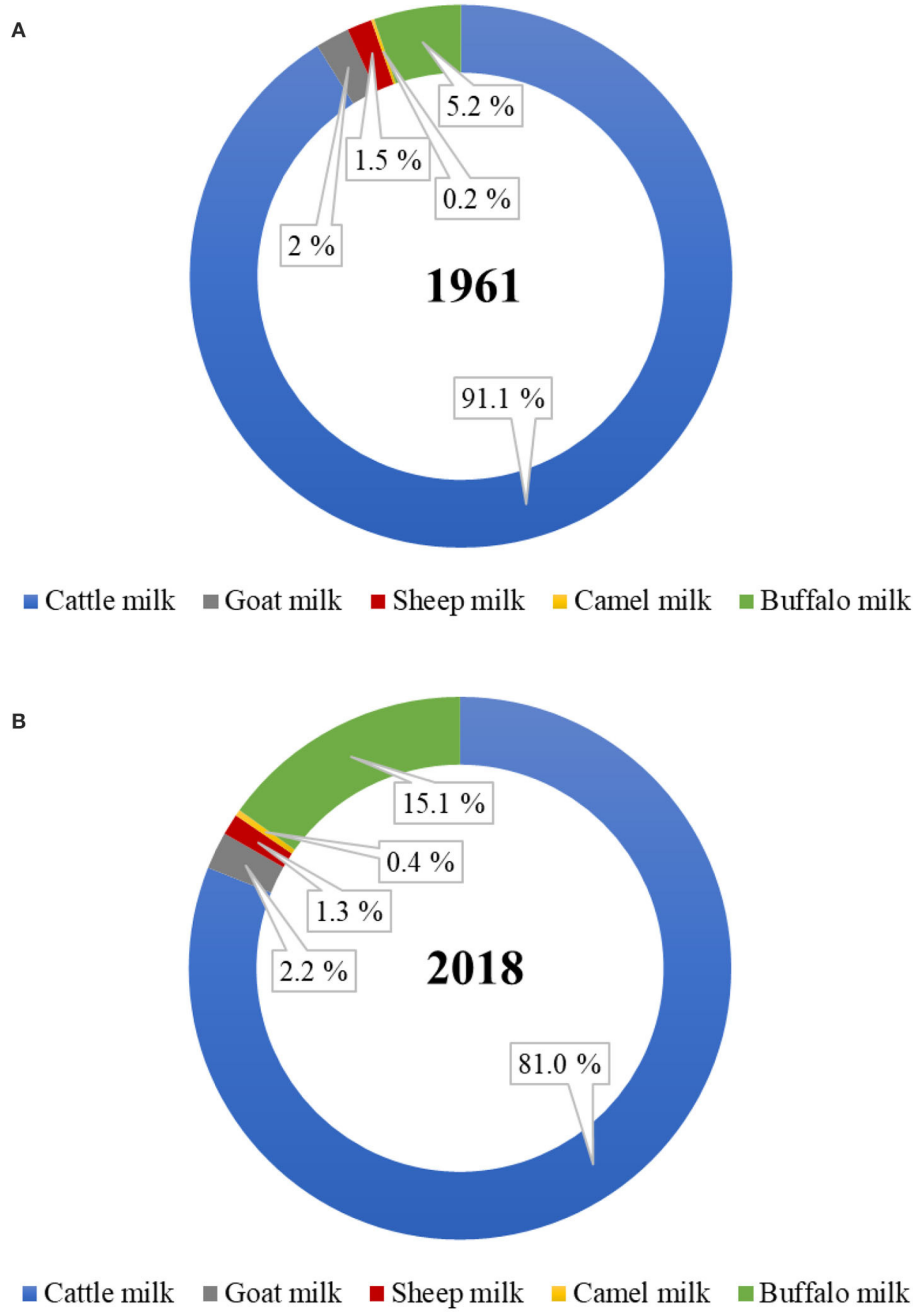
Proportion of dairy cattle and non-cattle milks produced globally in the year **(A)** 1961 and **(B)** 2018. (*Source:* FAOstat, March 2020).

Of the total world milk production, the proportion of total non-cattle milk production has increased from ~9% in 1961 to 19% in 2018 (Figure 1). Of the total global non-cattle milk production, buffalo milk has nearly tripled, camel milk has nearly doubled, and goat milk has slightly increased during this period. No world statistics on the amounts of milk produced from other dairy species, such as yak, horse, donkey, deer, musk ox, and llama, are available. Much of the non-cattle milk production remains officially unreported because of the unknown amounts that are consumed locally at a farmer's home or are sold directly by farmers to local people, especially in developing countries (6, 7).

The addition of milk as a product to non-cattle farm systems adds value and helps farmers in dealing with the fluctuating prices of meat, hair, and wool. The buffalo, goat, sheep, and camel milking industry is well set-up in many parts of the world, is gaining popularity, and is proving to be a profitable business for those who have already implemented it. Recently, New Zealand has introduced the development of a red deer dairy farming system. Large dairy companies as well as specialized small and medium enterprises (SMEs) are also interested in using non-cattle milks as a diversification strategy for their product portfolios. The regulatory requirements to ensure the safe production of cattle milk (and milk products) are well-defined in most of the world. However, the same regulatory limits may not be true to non-cattle milk and milk products. Thus, the emphasis on species-specific regulatory standards to guarantee the safety and quality of different milk for human consumption is needed (8–11). Also, understanding the significance of compliance to religious dietary laws (such as Kosher or Halal) will be of importance to the non-cattle milk-based dairy companies for gaining acceptance of their products from the various consumer groups (12).

In recent years, the opportunities for non-cattle milk production and the manufacture of products have expanded because the numbers of dairy cattle are perceived to be reaching their limit from environmental perspectives. Non-cattle milks are also believed to have certain nutritional benefits compared with cattle milks. For example, goat, sheep, camel, horse, and donkey milk are considered to be relatively more easily digestible, less allergenic, and more similar to human milk than cattle milk (4, 13, 14). In addition, non-cattle milks can be utilized for developing high value specialized dairy products of international as well as regional (local cultural) importance, such as cheese, yogurt, butter, ghee, ice-cream, fermented milk, probiotic dairy drinks, milk tablets, infant formulas (3, 15, 16). However, relatively little scientific information on the nutritional benefits of non-cattle milks is available. In addition, there is a significant gap in scientific knowledge on the detailed compositions, especially the minor components, and the protein and lipid structures in these milks.

## Comparative Compositions of Cattle and Non-cattle Milks

The comparative compositions of milk from different species have been extensively reviewed in previous studies (5, 17–19). The milk from different species vary in composition (Table 1). Protein, fat, lactose, and minerals are the four major components in all milks, irrespective of the species (18); the composition of milk within the same species varies considerably because of various factors, such as breed, stage of lactation, milking interval, type of feed, and climate (7, 19). For example, Li et al. (26) reported recently that the stage of lactation is a key factor responsible for differences in the compositional and physicochemical properties of dairy cattle milk in a seasonal calving system in New Zealand.

**Table 1:** General composition (g 100 mL^−1^) of milk from different mammalian species^#^.

	**Ruminants**						**Non-ruminants**		**Human**
**Properties**	**Cattle**	**Buffalo**	**Goat**	**Sheep**	Red deer^*^	**Camel**	**Horse**	**Donkey**	
Total solids	11.8–13.0	15.7–17.2	11.9–16.3	18.1–20.0	20.0–30.5	11.9–15.0	9.3–11.6	8.8–11.7	10.7–12.9
Protein	3.0–3.9	2.7–4.7	3.0–5.2	4.5–7.0	5.9–10.6	2.4–4.2	1.4–3.2	1.4–2.0	0.9–1.9
Fat	3.3–5.4	5.3–9.0	3.0–7.2	5.0–9.0	6.6-19.7	2.0–6.0	0.3–4.2	0.3–1.8	2.1–4.0
Lactose	4.4–5.6	3.2–4.9	3.2–5.0	4.1–5.9	2.6-6.2	3.5–5.1	5.6–7.2	5.8–7.4	6.3–7.0
Ash	0.7–0.8	0.8–0.9	0.7–0.9	0.8–1.0	1.04–1.18	0.69–0.9	0.3–0.5	0.3–0.5	0.2–0.3
Oligosaccharides^Ω^	0.003–0.006	No data	0.025–0.030	0.002–0.004	No data	No data	No data	No data	0.500–0.800

# *Source: Adapted and modified from Claeys et al. (19) and Crowley et al. (13)*.

* *Values based on minimum and maximum values found in the literature for different species of red deer; may include values from different stages of lactation (20–24)*.

Ω *Values derived from Martinez-Ferez et al. (25)*.

Non-ruminant milks (such as those from horse and donkey) are somewhat similar to human milk (in terms of protein, lactose, and ash contents), compared with dairy cattle milk and other ruminant milks (Table 1). Ruminant milks have higher protein and fat contents, compared with human milk and other non-ruminant milks. Human milk contains much higher amounts of total lactose-derived oligosaccharides than milk from other species (Table 1). Goat milk is also known to have a relatively higher oligosaccharide content, the composition of which is considered to be similar to that of human milk (27, 28).

### Proportions of Major Proteins

Compared with cattle milk and other ruminant milks, horse and donkey milk have a low casein-to-whey-protein ratio, similar to that in human milk. Among the ruminant milks, goat, sheep, and camel milk have a lower casein-to-whey-protein ratio as well as a relatively higher β-casein-to-α_s_-casein ratio compared with cattle milk (Table 2). Thus, these non-cattle milks are an attractive alternative as a potential natural ingredient for infant formula (13); a lower casein-to-whey-protein ratio (i.e., a higher proportion of whey proteins) has been shown to be more desirable for faster digestion of the milk proteins in infant formula than a casein-dominant protein composition (31, 32). As human milk has the lowest casein-to-whey-protein ratio, has a high β-casein-to-α_s_-casein ratio, and contains no β-lactoglobulin (Table 2), milk from other species with similar properties are of great interest to the consumer as well as to the dairy industry for the development of specialized dairy products, not only for infants but also for people in other age groups.

**Table 2:** Protein profile (g L^−1^) of milk from different mammalian species^#^.

	**Ruminants**						**Non-ruminants**		**Human**
**Protein fractions**	**Cattle**	**Buffalo**	**Goat**	**Sheep**	Red deer^*^	**Camel**	**Horse**	**Donkey**	
Total casein	24.6–28	32–40	23.3–46.3	41.8–52.6^∞^	~57–84	22.1–26.0	9.4–13.6	6.4–10.3	2.4–4.2
Total whey proteins	5.5–7.0	6	3.7–7.0	10.2–16.1^∞^	~11–15	5.9–8.1	7.4–9.1	4.9–8.0	6.2–8.3
Casein-to-whey protein ratio	82:18	82:18	78:22	76:24	~80:20–85:15	73:27–76:24	52:48	56:44	29:71–33:67
**Major caseins**									
α_s1_-Casein	8–10.7	8.9	0–13.0	2.4^∞^-22.1	–	4.9–5.7^Ω^	2.4	Present	0.77
α_s2_-Casein	2.8–3.4	5.1	2.3–11.6	6.0^∞^	–	2.1–2.5^Ω^	0.2	Present	Absent
β-Casein	8.6–9.3	12.6–20.9	0–29.6	15.6–39.6^∞^	–	14.4–16.9^Ω^	10.66	Present	3.87
κ-Casein	2.3–3.3	4.1–5.4	2.8–13.4	3.2–12.23^∞^	–	0.8–0.9^Ω^	0.24	Present	0.14
**Major whey proteins**									
β-Lactoglobulin	3.2–3.3	3.9	1.5–5.0	6.5–13.5^∞^	–	Absent	2.55	3.3	Absent
α-Lactalbumin	1.2–1.3	1.4	0.7–2.3	1–1.9	–	0.8–3.5	2.37	1.9	1.9–3.4

# *Source: Adapted and modified from Claeys et al. (19) and Crowley et al. (13)*.

* *Values (g kg ^-1^) derived from Arman et al. (20) and SDS-PAGE image analysis of our previous work (29). There are insufficient data for red deer milk in the literature to derive approximate values*.

Ω *Values derived from Kappeler (30)*.

∞ *Values (g kg ^-1^) derived from SDS-PAGE image analysis of our previous work (29)*.

β-Lactoglobulin is considered to be one of the major allergens that is responsible for cattle milk allergy in children (33). Thus, milk from species that lack β-lactoglobulin or have lower β-lactoglobulin-to-α-lactalbumin ratios are of interest for human consumption. Camel milk, like human milk, does not contain β-lactoglobulin (34, 35) or it may be present in trace amounts in different forms (36–38). Llama milk is also known to contain no β-lactoglobulin (5, 39), but little detailed information on its protein composition is available.

### Casein Micelle Characteristics

Individual caseins (α_s1_-, α_s2_-, β-, and κ-casein) are present in all milks as self-assembled particles known as “casein micelles” (40). The fundamental structure of the casein micelles in the milk from many species has not been studied in great detail, except in dairy cattle milk. Recently, Ingham et al. (41) used small-angle X-ray scattering and reported that the internal structures of the casein micelles of cattle, goat, and sheep milk had strong similarities with only slight differences, which may be due to the differences in casein composition, hydration, and physicochemical properties.

Apart from the differences in the proportions of different caseins (Table 2), the casein micelles in the milk from different species differ in size, hydration, and mineralization (Table 3). Among all mammalian milks, the casein micelles in human milk have the smallest diameter. The casein micelle sizes of goat, sheep, deer, camel, and horse milk are larger than that of human milk as well as cattle milk (Table 3). Sood et al. (53) reported that the loss of micellar calcium from the skim milk casein micelles (when dialyzed against same skim milk sample containing ethylenediaminetetraacetic acid, EDTA) resulted in increased hydration (or swelling) of casein micelles. Based on this, it was considered that the hydration level of the casein micelles was negatively correlated with mineralization of micelles (54) i.e., when the mineralization of the casein micelle increases, the degree of hydration of casein micelle decreases. Thus, the lower hydration of goat and sheep milk casein micelles had been related to its higher mineralization than those of cattle milk casein micelles (55, 56). Similarly, the casein micelles in buffalo milk (50) and donkey milk (51) are considered to be less hydrated and more mineralized than those in cattle milk.

**Table 3:** Casein characteristics of milk from different mammalian species^#^.

	**Ruminants**						**Non-ruminants**		**Human**
**Properties**	**Cattle**	**Buffalo**	**Goat**	**Sheep**	**Red deer**	**Camel**	**Horse**	**Donkey**	
Casein micelle	150–182	176^A^-180	180–301^B^	180–210	190^C^	380	255	100–200	64–80
diameter (nm)									
Hydration	1.92–3.7^D^	1.90^G^	1.43–2.05^F^	1.71–1.93^E^	No data	1.70^I^	No data	~1.0^H^	No data
(g H_2_O g protein ^−1^)									

# *Source: Adapted and modified from Claeys et al. (19)*.

*Values derived from other references are represented by uppercase case letters: A, Roy et al. (29); B, Nguyen et al. (42), Pierre et al. (43), and Pierre et al. (44); C, Roy et al. (29); D, Dalgleish (45), Wang et al. (46), and Dewan et al. (47); E, Pellegrini et al. (48); F, Remeuf et al. (49); G, Ahmad et al. (50); H, Luo et al. (51); I, Beaucher et al. (52)*.

It should be highlighted that there is a high degree of variation in the results that have been reported for the casein micelle characteristics within the same species, which may be due to differences in the analytical methods used. In addition, differences in breeds, genetic variants, and phosphorylation sites of the caseins may also add to the variation in the characteristics of the casein micelles within and across species (13).

### Milk Fat Composition

Compared with milk fat from other species (especially ruminants), human milk fat contains lower proportions of saturated fatty acids, higher proportions of monounsaturated fatty acids and polyunsaturated fatty acids, a higher ratio of ω-6 to ω-3 fatty acids, and higher levels of cholesterol (Table 4). In general, horse and donkey milk contain lower proportions of saturated fatty acids and higher proportions of polyunsaturated fatty acids than ruminant milks. In contrast, ruminant milks contain higher proportions of monounsaturated fatty acids, a higher ratio of ω-6 to ω-3 fatty acids, and a higher cholesterol content than horse and donkey milk (Table 4). The conjugated linoleic acid content is similar in human and ruminant milks but is lower in non-ruminant milks (Table 4).

**Table 4:** Fatty acid profile (% of total fatty acids) and cholesterol content of milk from different mammalian species^#^.

	**Ruminants**						**Non-ruminants**		**Human**
**Fatty acids**	**Cattle**	**Buffalo**	**Goat**	**Sheep**	**Red deer**	**Camel**	**Horse**	**Donkey**	
SFA (%)	55.7–72.8	62.1–74	59.9–73.7	57.5–74.6	No data	47–69.9	37.5–55.8	46.7–67.7	39.4–45
MUFA (%)	22.7–30.3	24.0–29.4	21.8–35.9	23.0–39.1	No data	28.1–31.1	18.9–36.2	15.31–35.0	33.2–45.1
PUFA (%)	2.4–6.3	2.3–3.9	2.6–5.6	2.5–7.3	No data	1.8–11.1	12.8 −51.3	14.17–30.5	8.1–19.1
ω-6: ω-3 fatty acids ratio	2.1–3.7	No data	4	1.0–3.8	No data	No data	0.3–3.5	0.9–6.1	7.4–8.1
CLA (%)	0.2–2.4	0.4–1	0.3–1.2	0.6–1.1	No data	0.4–1	0.02–0.1	No data	0.2–1.1
Cholesterol (mg/100 mL milk)	13.1–31.4	4–18.0	10.7–18.1	14–29.0	No data	31.3–37.1	5.0–8.8	No data	14–20
% of C16:0 at sn-2	38	37	36	29	No data	No data	No data	54	74

# *Source: Adapted and modified from Claeys et al. (19) and Crowley et al. (13)*.

*SFA, saturated fatty acids; MUFA, monounsaturated fatty acids; PUFA, polyunsaturated fatty acids; CLA, conjugated linoleic acid, C16:0, palmitic acid on the sn-2 position of milk TAG*.

Sheep and goat milk fats are known to be rich in short chain (responsible for the distinct flavor of these milks) and medium chain triacylglycerols (TAGs); similarly, buffalo milk fat contains higher proportions of medium chain TAGs than cattle milk, which has high proportions of long chain TAGs (57–60). In contrast, camel milk contains a higher proportion of long chain fatty acids and a lower proportion of short chain fatty acids than cattle milk (61). Data for the fat composition of red deer milk are scarce, but this milk is considered to contain 5–10% fewer unsaturated fatty acids and higher proportions of shorter chain and saturated fatty acids than cattle milk (21). These differences may contribute to the different digestion behaviors of the milk fat from different species, as short or medium chain TAGs are considered to be more efficiently hydrolyzed by lipases (62, 63).

Free long chain saturated fatty acids, such as palmitic acid (C16:0), are not considered to be efficiently absorbed in the body as they form insoluble fatty soaps with calcium in the small intestine (64, 65). In this context, the TAG structure is considered to play a key role. Most of the long chain palmitic acid (C16:0) present in human milk (>70%) is located in the sn-2 position of the TAG structure; this position is considered to be suitable for the digestion and absorption of this fatty acid as well as other nutrients (18, 62, 66). German and Dillard (64) stated that the location of saturated fatty acids, such as long chain palmitic acid on the sn-2 position of TAGs, makes both the sn-1 and the sn-3 position fatty acids easily hydrolyzable by pancreatic lipases into free fatty acids, and produces sn-2 monoacylglycerols, which are easily absorbed in the small intestine; this also makes the milk calcium completely available and absorbable. Donkey milk has the closest proportion of palmitic acid located at the sn-2 position (i.e., 54%) to that of human milk (74%) (Table 4). Thus, the modification of the TAG structure in milk from other species may help to deliver better milk fat digestion profiles; this could be an area of future interest.

### Milk Fat Globule Size

The fat in the milk of all species is present as small spherical droplets, called globules, the diameter of which ranges from 0.2 to 15 μm (67). The size of these fat globules varies among milk from different species; goat, sheep, camel, and equine (horse and donkey) milk have higher proportions of smaller size fat globules compared to cattle milk (Table 5). The differences in the sizes of the fat globules of milk from different species may influence the digestion of their fat differently (18, 19). The TAG core of the fat globules from all species is surrounded, protected, and stabilized by a phospholipid trilayer (along with specific membrane proteins) called the milk fat globule membrane (MFGM) (68, 69). The MFGM is unique to milk and its structure is considered to be similar in all milks, although the proportions of different proteins in the MFGM may differ among different species (70).

**Table 5:** Fat globule size of milk from different mammalian species^#^.

	**Ruminants**						**Nonruminants**		**Human**
**Property**	**Cattle**	**Buffalo**	**Goat**	**Sheep**	**Red deer**	**Camel**	**Horse**	**Donkey**	
Fat globule diameter (μm)	2.8–4.6	4.1–8.7	2.6–3.7^A^	3.0–4.6	2.7–6.6^A^	3.0	2–3	1–10	4

# *Source: Adapted and modified from Claeys et al. (19) and Crowley et al. (13)*.

*Values derived from other references are represented by uppercase case letters: A, Roy et al. (29)*.

In general, the differences in the characteristics of the casein micelles and the fat globules among different milks are considered to play important roles in influencing their coagulation behavior and nutrient delivery during digestion, which is discussed in the section on milk digestion.

## Hypoallergenic Potential of Non-cattle Milks

More than 20 proteins in cattle milk are known to cause allergic reactions; of these, the casein fractions (especially α_s2_-, α_s1_-, and κ-caseins as well as, to some extent, β-casein), lactoferrin, serum albumin, and β-lactoglobulin are considered to be the most common cattle milk allergens (71–73).

There is increasing interest with respect to the suitability of non-cattle milks as a hypoallergenic option to cattle milk (74). A few studies have reported that horse milk (75), donkey milk (76, 77), camel milk (78, 79), and water buffalo milk (80) may be potential alternatives in cases of moderate allergenicity to cattle milk in children; however, this needs to be further investigated because weak cross-reactivity of non-cattle milk proteins with cattle milk proteins has been reported (81–83). Jenkins et al. (71) conducted a comprehensive study on the cross-reactivity of human and non-human milk proteins and found that the degree of allergenicity of a non-human milk protein is related to its extent of similarity with its human homologs. They found that, compared with cattle, goat, and sheep milk proteins, camel and horse milk proteins (i.e., α_s1_- and β-caseins) are more homologous to their human milk counterparts, which may be a reason for their weak cross-reactivity or less allergenic nature compared with other non-cattle milks.

Infante et al. (84) reported that 25% of patients had a negative immunological test for adverse reactions to goat milk proteins; thus, goat milk cannot be considered to be a suitable alternative in cases of cattle milk allergy. Similarly, there is also strong evidence of allergenicity or positive cross-reactivities of goat, sheep, deer, and buffalo milk with cattle milk (83, 85–87). In addition, reports concerning selective allergy to goat and sheep milk proteins, but not to cattle milk proteins, are also available (88, 89). Bevilacqua et al. (90) found that goat milk with lower proportions of αs_1_-casein (and higher amounts of αs_2_-casein) was significantly less allergenic in guinea pigs than goat milk with high α_s1_-casein content (and low α_s2_-casein content); thus, different proportions of milk proteins may also play a key role in controlling milk protein allergy.

Overall, the scientific evidence indicates that there is little basis for promoting non-cattle milk or milk proteins as an alternative to cattle milk for people suffering from cattle (or cow) milk allergy.

## Milk Digestion

### Indispensable Role of the Gastric Phase in Milk Digestion

It is well-accepted that milk is a source of nutritionally balanced and highly digestible proteins (91, 92). Previous studies have reported that the gastric emptying rates of two major fractions of milk protein (i.e., casein and whey protein) differ markedly; this has led to the concept of “slow” digested caseins and “fast” digested whey proteins (93–98).

The digestion of milk by the stomach enzymes (mainly pepsin and, to some extent, gastric lipases) in the presence of hydrochloric acid is considered to be the first key step, which is followed by further digestion in the small intestine by intestinal proteases and lipases (99, 100). Some human infants may have chymosin like enzyme along with pepsin, which disappears from the gastric fluid by day 11 after birth (101). Chymosin and pepsin belong to the same group of aspartic proteinases that uses aspartic acid residues in their active center (102). Both the enzymes can preferentially hydrolyze the Phe105–Met106 bond of κ-casein, except that pepsin also exhibits unspecific proteolytic activity toward bonds with Trp, Tyr, Leu or Val residues, and thus have higher proteolytic activity relative to its milk clotting activity than chymosin (102–104). As the site of action of both chymosin and pepsin is the same, the mechanism of action of chymosin and pepsin is expected to be similar in relation to milk clotting. Chymosin is most stable in the pH range 5.3–6.3, but loses its activity rapidly under acidic conditions, i.e., below pH 3–4, as well as at high alkaline pH values, i.e., above pH 9.8 (105). Pepsin has maximum proteolytic activity at pH 2, with an optimum pH range of 2–5, and has activity in the pH range pH 5.5–7.5. Pepsin is irreversibly inactivated at pHs above 7.5 (106).

The protein hydrolysis sites of pepsin are different from those of the intestinal proteases (mainly trypsin and chymotrypsin). Pepsin acts preferentially on κ-casein on the casein micelles, leading to the coagulation of the casein fraction of milk proteins under acidic conditions, whereas the whey protein fraction remains soluble (107). Thus, the early role played by the stomach in milk digestion is an essential step in regulating the rate of digestion of the milk proteins in the gastrointestinal tract (108). In this respect, it is of great importance to understand the digestive dynamics and coagulation behavior of milk during gastric digestion, as milk coagulation can influence the delivery rates of proteins, fats, and associated milk constituents.

### Evidence of Milk Coagulation

Human milk is known to form very soft and fragile curds in the infant stomach. Mason (109) investigated the changes in pH and the extent of protein hydrolysis in the stomach contents collected using a gastric tube at different time intervals from 25 healthy newborn infants (full-term, aged between 5 and 13 days). He reported the presence of casein curds in the stomach contents collected after 30 min of breastfeeding. He also reported that there was negligible protein hydrolysis in these samples. Similarly, recently, de Oliveira et al. (110) studied the gastric digestion of raw and pasteurized human milk in tube-fed preterm infants. The microstructural analysis in their study showed that human milk formed very soft and fragile protein aggregates in the infant's stomach.

Piglets and growing pigs have been regarded as a suitable animal model for human digestion research (111–113). Bottle-fed piglets have been used to study the digestion of human milk and infant formulas (114–116). Some evidence of clot (or curd) formation by cattle milk in pigs or piglets has been reported in the literature. Washburn and Jones (117) reported that cattle skim milk formed a tough or hard clot, whereas cattle whole milk formed a more friable and mellow curd in the stomach of baby pigs (28–35 days old), and that, the higher the fat content, the softer was the curd that formed. Braude et al. (118) found that the caseins from homogenized cattle milk clotted in the stomach of the 28-day-old pig after 15–30 min of feeding, whereas the “whey” fraction of the milk remained soluble and passed rapidly into the small intestine. Similarly, Decuypere et al. (119) reported the formation of firm casein clots in the stomachs of early weaned pigs (10–29 days of age) fed dry cattle-milk-based food; their gastric chyme had a long retention time and a low buffering capacity and stimulated more gastrin release, compared with the gastric contents of suckling piglets fed pig milk. They believed that these differences were due to the firm casein clot formed by a dry cattle-milk-based food in early weaned pigs in comparison with the soft casein aggregate formed from pig milk in suckling piglets.

### Clotting Characteristics of Human Milk and Cattle Milk and Its Implications

Cattle milk is known to form firm curds (or clots) in the stomach, in comparison with human milk.

Nakai and Li-Chan (108) studied the coagulation characteristics of human and cattle milk using an *in vitro* acid precipitation test at 37°C, in which they added 0.2% acidic pepsin solution to 100 mL each of cattle milk and human milk at a flow rate of 15 mL/h. They found that human milk formed much finer protein aggregates (or clots) than cattle milk and reported that this could be the possible reason for the shorter gastric emptying time for human milk.

The differences in the structures of human and cattle milk curds could be related to the differences in their fat and protein compositions. The protein (casein)-to-fat ratio of human milk is very low (Tables 1, 2) compared with that of cattle milk (as well as of other non-cattle milks), which is likely to be a factor that is responsible for its soft (or fragile) curd formation. In addition, the higher β-casein-to-α_s_-casein ratio of human milk has been associated with the fine and loose curd formed by human milk in an infant stomach. Lichan and Nakai (120) performed an *in vitro* coagulation study with untreated cattle milk casein, rennin-modified cattle milk casein, and human milk casein. The rennin-modified cattle milk casein was a β-casein-rich cattle milk (similar to β-casein-rich human milk) that was produced by selectively eliminating the α_s1_-casein fraction from cattle milk by a process involving rennet action. Upon acidification of the different casein solutions to pH 2 or pH 4, Lichan and Nakai (120) observed that the hardness of the clot formed from these different casein solutions decreased in the order: cattle milk casein > rennin-modified cattle milk casein (rich in β-casein) > human casein. In another study, Lichan and Nakai (121) also reported that moderate or partial dephosphorylation of cattle milk casein using different phosphatases (calf intestinal alkaline phosphatase and potato acid phosphatase) at pH 4 resulted in the acid-coagulating properties of these modified cattle milk casein solutions being similar to those of human milk as well as in a greater rate of proteolysis compared with the firm clots of untreated cattle milk casein. However, all these studies were *in vitro* physicochemical studies, and further studies in *in vitro* or *in vivo* digestion models need to be conducted to validate such findings.

Blakeborough et al. (122) studied the digestion of human milk, cattle milk, and reconstituted baby formula (based on full cream dry cattle milk powder) using 14-day-old piglets; cattle milk or baby formula formed firm solid curds, whereas human milk formed a very liquid-like coagulum (little solid material) in the piglet's upper gastrointestinal tract. They also determined the bioavailability of zinc (Zn) from these milk systems; they found that, for cattle milk (as well as baby formula), ~55–72 and ~60–66% of the Zn was retained in the curds present in the gastric chyme and the intestinal digesta, respectively, whereas, for human milk, ~43 and 7% of the Zn was retained in the curds present in the gastric chyme and the intestinal digesta, respectively. They suggested that these differences in the distribution and bioaccessibility of Zn in the gastrointestinal tract of piglets fed human milk or cattle milk may have been due to the differences found in the consistency of the casein curds formed by the different milks.

### Digestion of Milk From Different Species

#### Protein Digestion

The lower protein content, lower casein-to-whey-protein ratio, and higher β-casein-to-α_s_-casein ratio of human milk compared with milk from other species have been related to its soft curdling properties *in vitro* as well as *in vivo*, as described earlier. Although none of the non-human milks match the composition of human milk, horse, and donkey milk are known to form very weak or fragile gels (or curds or flocs) when acidified or treated with rennet (123–125) and thus are expected to form soft or fragile curds in the stomach, in comparison with cattle milk, because of their lower casein content. Similarly, some of the ruminant milks, such as goat and camel milk (126–130), are also considered to form soft curds in the stomach when acidified or treated with rennet (or pepsin), because of their lower casein content or larger casein micelle size compared with cattle milk, even though they contain comparatively higher proportions of caseins than equine and human milk. However, no direct comparative *in vitro* or *in vivo* digestion studies between cattle and non-cattle milks, focusing on their curd formation characteristics in the stomach, have been reported to date. There are only a few comparative *in vitro* digestion studies on cattle and non-cattle milks, focusing on their protein or fat digestion.

Jasińska (131) compared the degrees of hydrolysis by pepsin and trypsin of micellar caseins obtained from cattle, human, goat, and horse skim milk; the peptic hydrolysis rates of the micellar caseins from cattle, human, goat, and horse milks were 23–42 (differed for different breeds of cattle), 80, 65, and 43%, respectively. The tryptic hydrolysis rates of the micellar caseins from cattle, human, goat, and horse milk were 76–90, 100, 96, and 92%, respectively. The higher susceptibility of human and goat milk was believed to be due to the smaller micellar aggregates and the presence of higher proportions of β-casein in their micellar structures, when compared with cattle milk (which had higher proportions of α_s1_-casein).

Recently, Hodgkinson et al. (132) studied the *in vitro* static gastric digestion of cattle and goat whole milk (at pH 3.0) and reported that, after both 20 and 60 min of digestion, goat milk caseins were digested faster than cattle milk caseins (based on sodium dodecyl sulfate polyacrylamide gel electrophoresis (SDS-PAGE) image analysis), possibly because of the relatively soft or fragile coagulum formed by goat milk. Tagliazucchi et al. (133) also studied the *in vitro* static gastrointestinal digestion of cattle, goat, sheep, and camel skim milk (as per the INFOGEST protocol) and reported that the extent of free amino groups generated during the gastric digestion was higher for goat, sheep, and camel milk proteins, indicating that the proteins in these non-cattle milks were hydrolyzed faster than cattle milk proteins during the gastric step. However, after the intestinal step, they reported that only the goat milk proteins were hydrolyzed faster than the milk proteins from the other species, all of which had similar hydrolysis rates. Tagliazucchi et al. (134) and Rutella et al. (135) reported similar findings in their previous studies, i.e., that the degree of hydrolysis of goat skim milk proteins during the gastric and intestinal steps was much higher than that of cattle skim milk proteins. The authors stated that the higher degree of hydrolysis of goat milk proteins observed in all studies was probably due to the higher susceptibility of goat milk proteins to pepsin.

Maathuis et al. (136) investigated the comparative protein digestibilities and qualities (based on bioaccessible nitrogen and amino acids) of human milk, cattle-milk-based infant formula, and goat-milk-based infant formula using the tiny-TIM model (a dynamic *in vitro* infant gastrointestinal model). They found that the protein digestibilities and qualities of all diets were similar; however, the rates of protein digestion were slower during the first 60 min of digestion for the cattle-milk-based formula than for the human milk and the goat-milk-based formula. They hypothesized that the differences in the clotting characteristics of different milks would have led to differences in their gastric emptying, as they found that the curds formed from the cattle-milk-based formula were retained for a longer duration in the gastric compartment of tiny-TIM compared with those from the human milk and the goat-milk-based infant formula. Similarly, Ye et al. (32) investigated the *in vitro* dynamic gastric digestion of goat- and cattle-milk-based formulas in a mini version of the human gastric simulator (HGS), simulating infant gastric digestion. The authors found that the goat-milk-based infant formula formed smaller protein aggregates in the mini-HGS, leading to faster hydrolysis of its proteins compared with those from the cattle milk formula. Based on the above-mentioned studies it appears that the differences in the structures of the curds formed from milk of different species during gastric digestion may be a key factor that is responsible for their different digestion behaviors.

In contrast, Almaas et al. (137) did not find any differences in the digestion of caseins and α-lactalbumin from cattle and goat skim milk (with high and low αs_1_-casein content) after static gastrointestinal digestion using human gastric juice (HGJ) and human duodenal juice (HDJ). They also did not find any differences between goat milk with high and low αs_1_-casein content after digestion with HGJ and HDJ. However, they observed (using SDS-PAGE image analysis) that goat milk β-lactoglobulin was rapidly digested during both gastric digestion and intestinal digestion, compared with cattle milk β-lactoglobulin. El-Zahar et al. (138) studied the hydrolysis of isolated β-lactoglobulin from sheep and cattle milk by porcine pepsin and found that β-lactoglobulin from sheep milk was hydrolyzed faster because of its slightly different tertiary structure and higher surface hydrophobicity. As β-lactoglobulin is considered to be one of the major allergens (as it is absent in human milk), the higher degree of hydrolysis by pepsin of the β-lactoglobulin in goat and sheep milk may be a possible reason that these non-cattle milks are better tolerated by some people than cattle milk.

Vithana et al. (23) studied the comparative *in vitro* gastrointestinal digestion of raw cattle and deer skim milk. They found that, after gastric digestion, nearly 49 and 27% of the deer and cattle milk caseins remained undigested (SDS-PAGE image analysis), respectively, whereas, after intestinal digestion, the caseins from both species were completely digested. This, indicated that, during the gastrointestinal digestion, deer milk caseins were digested at a faster rate than cattle milk caseins. We hypothesize that the higher amounts of caseins retained in the gastric phase for deer skim milk may have been due to the higher protein content (as well as casein content) of the deer milk used in their study, indicating that the inherent composition of milk also has a key role to play during gastric digestion. Vithana et al. (23) also found that α-lactalbumin was hydrolyzed faster in deer milk than in cattle milk. However, β-lactoglobulin from both species was found to be resistant to both gastric and duodenal digestion.

In contrast to the above studies, some studies have reported no differences or faster hydrolysis of cattle milk proteins than of goat milk proteins. For instance, Inglingstad et al. (139) reported (based on SDS-PAGE image analysis) that 69 and 82% of the caseins remained undigested after hydrolysis by HGJ of cattle and goat skim milks respectively; however, after further treatment with HDJ, almost all of the caseins from the milk of both species were digested. They found that the β-lactoglobulin and α-lactalbumin from both species were highly resistant to HGJ and that, after hydrolysis with HDJ, ~64% of the β-lactoglobulin from both species remained undigested and 91 and 65% of the α-lactalbumin from the cattle and goat skim milk respectively, remained undigested. Mros et al. (140) reported no differences in the protein digestion of cattle, goat, and sheep skim milk following hydrolysis by pepsin and pancreatin.

Similarly, Milan et al. (141) reported that whole goat-protein fortified milk, compared to whole cow-protein fortified milk, was digested and metabolized similarly (despite the differences in their inherent nutrient composition) in young adults (aged 18–28 years). However, they dissolved paracetamol in fortified milk drinks before giving it to the participants for consumption (plasma paracetamol levels were used as a marker for gastric emptying). It has to be noted that depending on the type of paracetamol used, it may have a buffering action during the gastric digestion in the stomach (142) and thus, careful consideration needs to be made while conducting human digestion studies to draw any firm conclusions.

Vaisman et al. (143) investigated the gastric emptying times in humans of camel and cattle milk using a scintigraphic technique and reported that the poor coagulation properties of camel milk (as observed during acid or rennet coagulation) did not provide any comparative advantage over cattle milk in terms of gastric emptying. It should be noted that the soft or fragile curd formed from non-cattle milks (such as camel, goat, horse, and donkey milk) during acid or rennet coagulation provides only an indication of how these non-cattle milks may behave in the human stomach during gastric digestion. The gastric digestion process is a complex and dynamic phenomenon, and in-depth comparative *in vitro* and *in vivo* studies on cattle and non-cattle milks that simulate the gastric digestion in humans need to be undertaken, to draw any definite conclusions.

**Figure 2:**
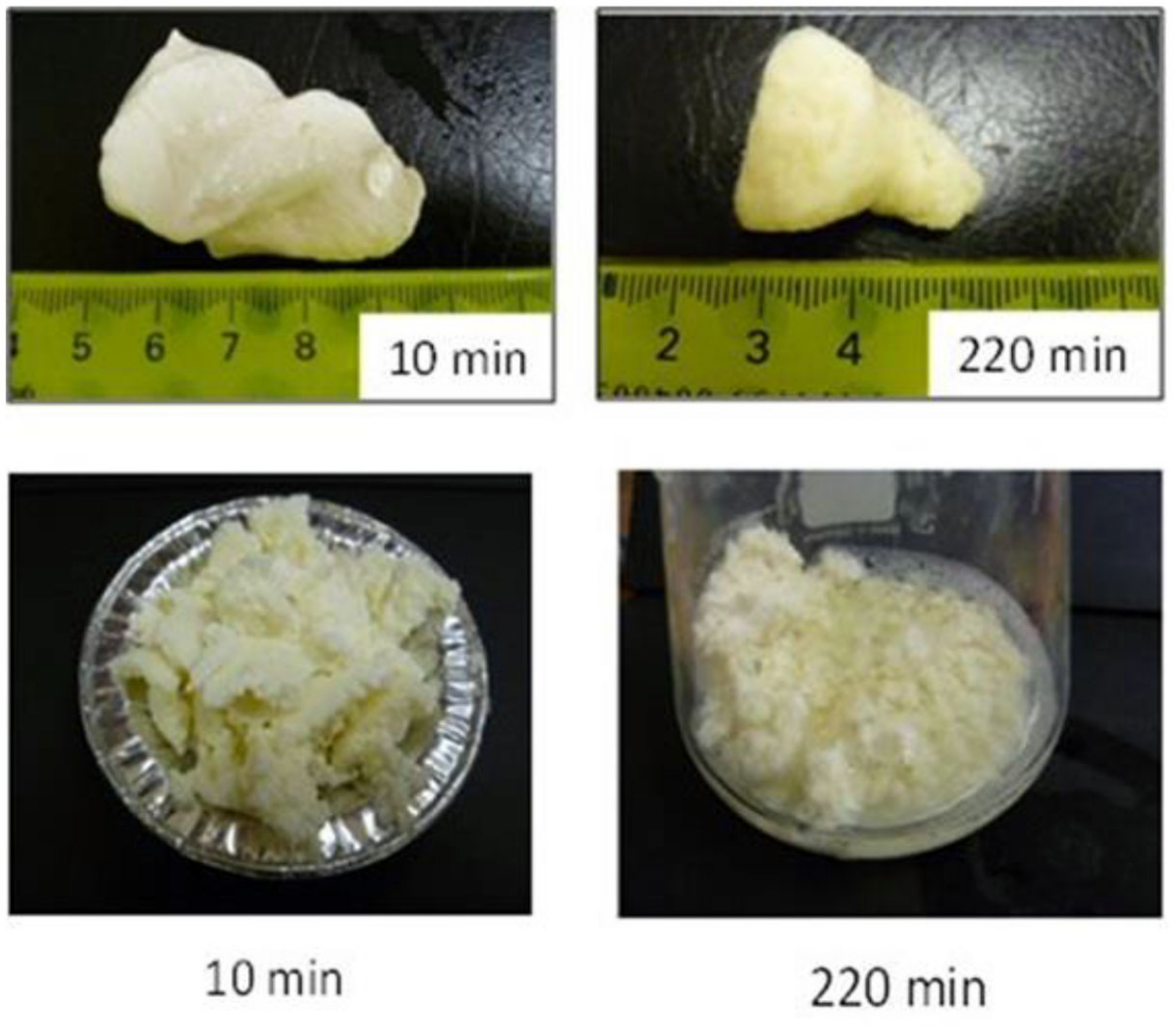
Images of clots formed during the gastric digestion of 200 g of unheated (top row) and heated (bottom row) cattle skim milk at different digestion times. *Source:* Adapted from Ye et al. (107).

**Figure 3:**
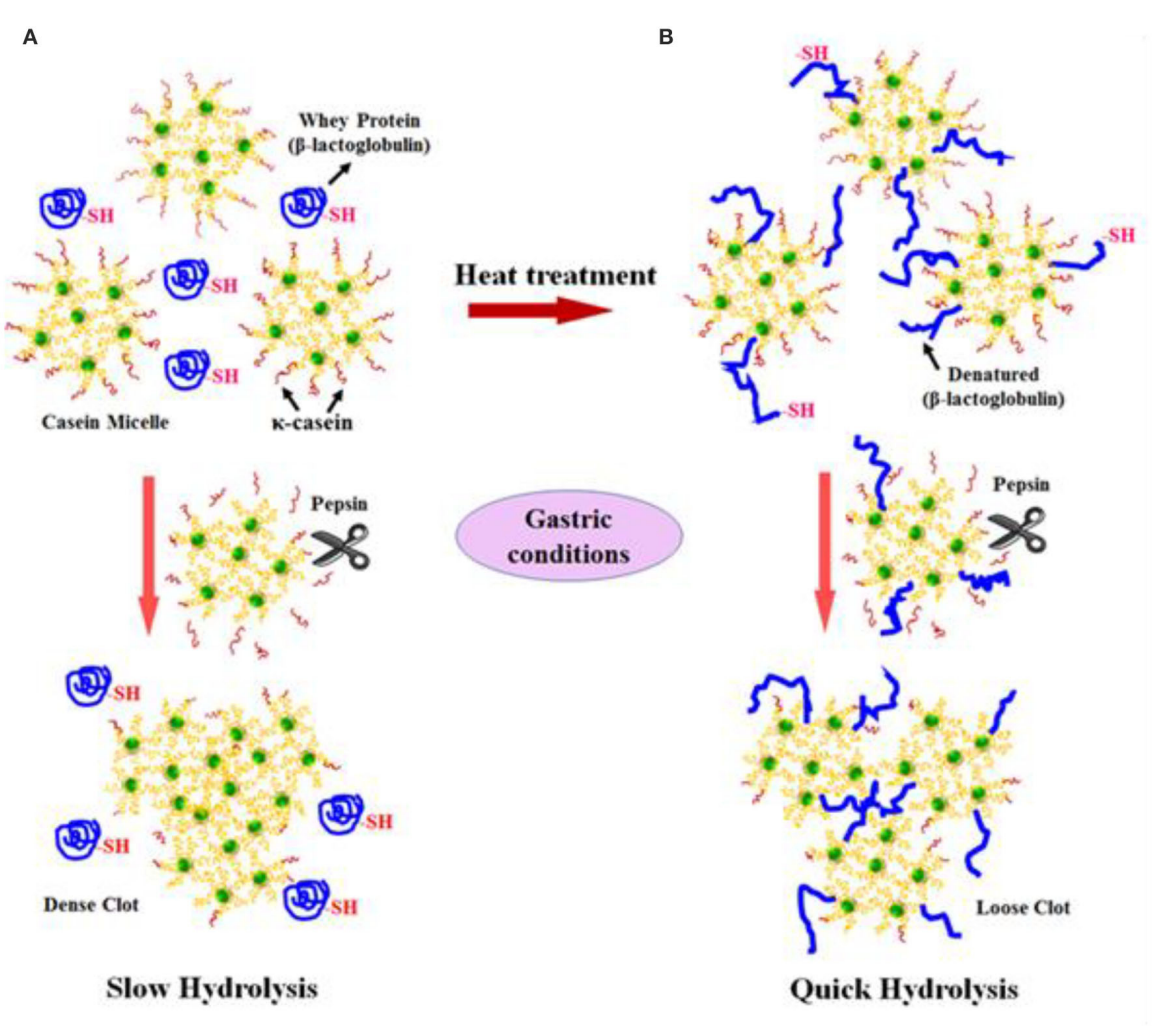
Schematic diagram of the possible mechanism of events during the formation of protein curds from **(A)** raw milk (unheated) and **(B)** heated milk during gastric digestion. *Source:* Adapted from Ye et al. (145).

Not only protein composition and (or) casein micelle structure, but also different processing temperature and time combinations may induce differences in the curd structure in the stomach, which may influence the rate of delivery of proteins to the small intestine and their subsequent absorption. For instance, Ye et al. (107) studied the dynamic gastric digestion behavior of raw and heated (90°C for 20 min) cattle skim milk using an HGS. The HGS is a dynamic stomach model that is capable of simulating the stomach contraction forces and the flow of gastric fluids that occur *in vivo* (144). Ye et al. (107) found that raw milk formed a “closely knitted” tight clot, whereas heated milk formed fine and loose protein aggregates (Figure 2), leading to slow hydrolysis of caseins from raw milk, compared with heated milk. This was because, in raw milk, only the caseins were involved in clot formation, whereas, in heated milk, both the caseins and denatured whey proteins were involved in clot formation (145). Heating at 90°C for 20 min would have led to complex formation between fully denatured whey proteins and caseins via sulfhydryl groups and disulfide linkages (Figure 3), hindering the formation of a firm clot (146, 147). Kaufmann (148) reported that ultrahigh-temperature-treated (UHT) milk led to the formation of soft coagulates in the mini-pigs stomach, leading to higher levels of amino acids and urea in their blood serum compared to that of pasteurized and raw milk, which formed stronger coagulum. Thus, these differences in gastric restructuring induced by heating are expected to be a key possible reason for higher postprandial utilization of dietary nitrogen from defatted UHT milk (140°C for 5 s) compared to defatted pasteurized milk (72°C for 20 s) as well as defatted microfiltered milk in humans (149).

Doan (150) published a comprehensive review based on studies on the gastric digestion of processed (boiled, evaporated, or acidified) and raw cattle milk in the early 1900s, and reported that boiled, evaporated, or acidified milk were emptied rapidly from the human stomach because of the finer or softer curd that formed. It was suggested that the modification of raw cattle milk using different processing conditions may be a potential option in the development of dairy-based baby foods or beverages with properties similar to those of human milk.

To date, no studies on the impact of different heating or processing conditions on the digestion behaviors of non-cattle milks have been reported in the literature. It should be noted that the commercial processing or technological conditions needed for non-cattle milks may be different from those needed for cattle milk. In addition, the impact of different processing conditions on the digestion behaviors of non-cattle milks may be different from that on cattle milk because of the differences in their composition and structures.

### The Influence of the Protein Network on Fat Digestion—The Whole Milk Matrix

During the gastric digestion of whole milk, the fat globules are known to be physically entrapped within the protein clot that is formed. Thus, the nature or structure of the protein network formed will influence the rate of release and the digestion of fat by gastrointestinal lipases (145, 151–153). Previous studies have shown that the nature or structure of the protein network formed is, in turn, dependent on the protein composition (casein-to-whey-protein ratio), the protein-to-fat ratio, and the impact of different processing conditions (99, 154). For instance, Mulet-Cabero et al. (154) studied the *in vitro* gastrointestinal digestion of model systems based on different casein-to-whey-protein ratios using a semidynamic gastric model, and reported that the viscosity or firmness of the coagulum formed increased as the casein-to-whey-protein ratio increased in the model protein systems, leading to slower gastric emptying, and slower digestion and absorption of nutrients. They also found that the addition of increasing amounts of fat to the casein-rich protein models produced more fragmented clots with a significant decrease in their firmness. This, indicates that the presence of fat hindered the aggregation of proteins, which may, in turn, influence the digestion rates of nutrients.

**Figure 4:**
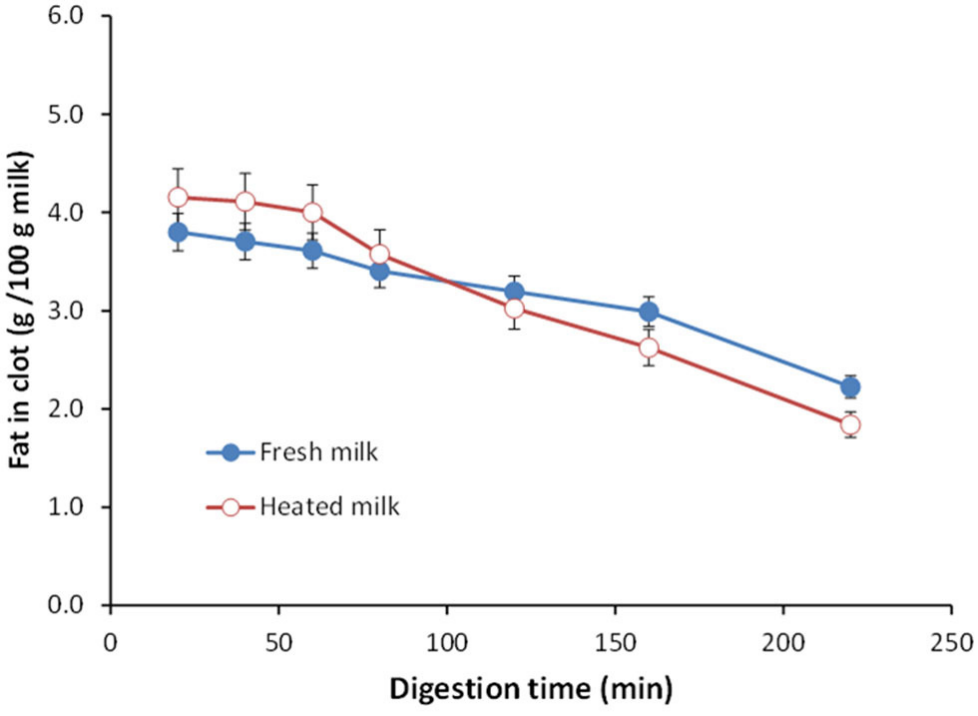
Changes in the fat content (g/100 g milk) in clots obtained from (•) unheated (raw) and (°) heated cattle whole milk during gastric digestion. *Source:* Adapted from Ye et al. (151).

Ye et al. (151) studied the gastric digestion of raw (unheated) and heated (90°C for 20 min) cattle whole milk and reported that the release of fat globules was dependent on the disintegration characteristics of the protein clot and that the release of fat globules was higher from the finer aggregates of protein clots formed from the heated whole milk than from the firm clots formed from the raw whole milk (Figure 4). Similarly, Ye et al. (145) studied the comparative *in vitro* and *in vivo* (in rats) gastric digestions of raw (nonhomogenized), pasteurized (homogenized), and UHT (homogenized) cattle whole milk, and reported that the UHT milk had faster rates of protein hydrolysis as well as release of fat globules during gastric digestion, compared with the raw and pasteurized milk; the differences were attributed to the smaller or fragmented protein aggregates formed from the UHT milk proteins in comparison with the aggregates from the other milks.

**Figure 5:**
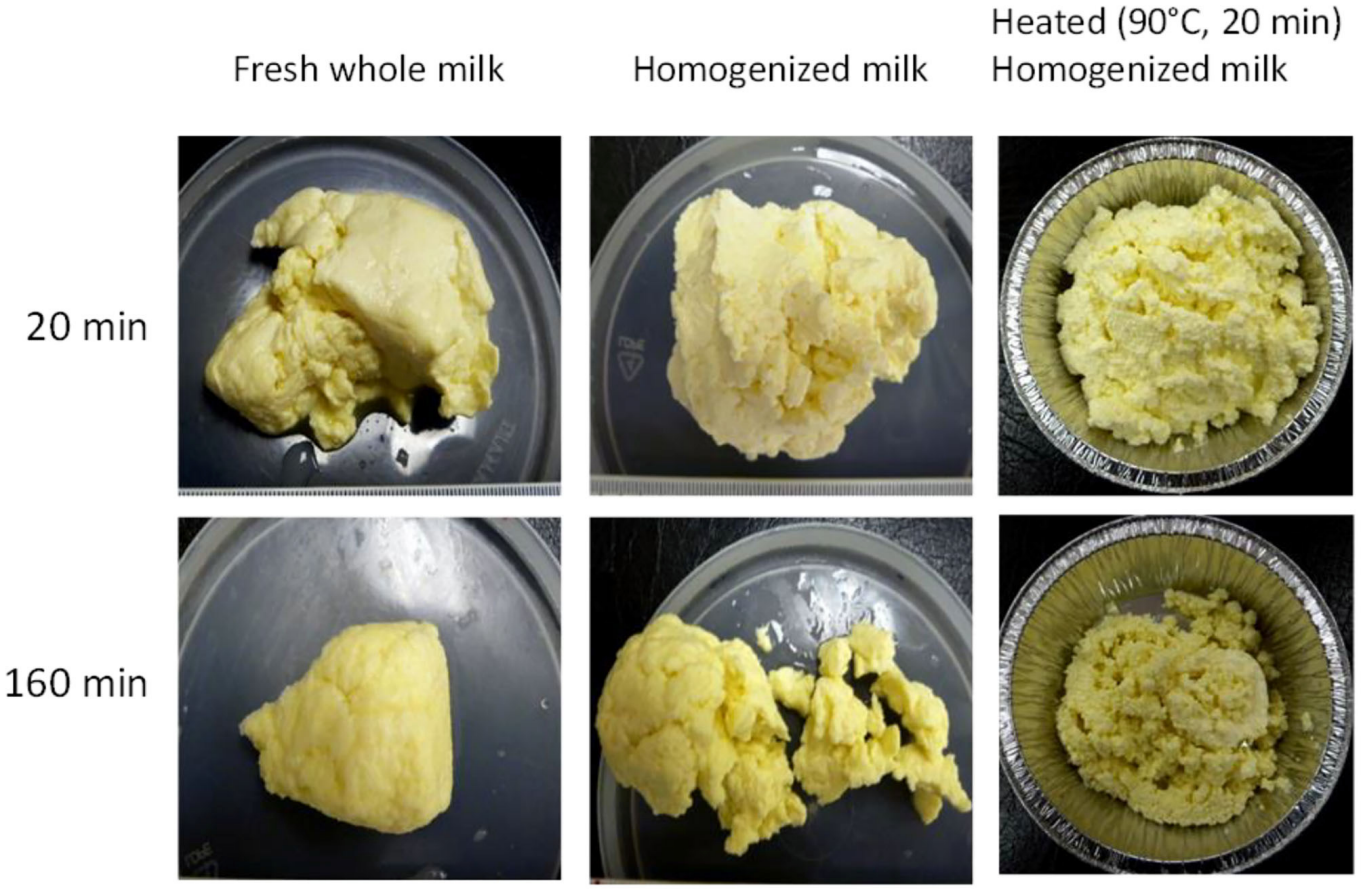
Images of clots formed during the gastric digestion of raw (unheated), homogenized, and heated homogenized cattle whole milk during 20 and 160 min of gastric digestion. *Source:* Adapted from Ye et al. (152).

In another gastric digestion study, Ye et al. (152) reported that the release of fat globules was relatively higher in homogenized milk (20/5 MPa (primary/secondary pressure), 20°C) as well as heated, homogenized milk (20/5 MPa, 20°C + 90°C for 20 min) because of the fine and crumbled structure of the coagulum formed in these milks compared with the firm coagulum formed from raw cattle whole milk (Figure 5). Similar results have been reported by Mulet-Cabero et al. (153) for processed cattle whole milks.

The coalescence of fat globules entrapped within the protein network as well as those present in the liquid phase of the gastric chyme has also been reported in the above-mentioned studies (145, 152, 153), which is expected to be due to the hydrolysis of the proteins present at the surface of the milk fat globule (present naturally in the MFGM or adsorbed proteins because of processing treatments), leading to destabilization of the fat globules and coalescence.

As the milk from different species are known to vary in fat content, protein-to-fat ratio, fat globule size, and structure, there may be differences in the consistency of the coagulum formed from milk of different species during gastric digestion, which may impact their overall digestion behavior differently.

### Gastrointestinal Digestion of Fat

Little information is available on the gastric digestion of milk fat, irrespective of species. Lipolysis during the gastric phase was previously considered to be of less relevance during the overall digestion process as gastric lipolysis accounts for only 10–25% of the overall lipid digestion in adults (155). Therefore, most of the studies reported in the literature on fat digestion have focused mainly on intestinal digestion. However, it is now widely suggested that gastric lipases should be incorporated in *in vitro* digestion studies as their preliminary role may facilitate further breakdown of lipids by intestinal lipases (155). Also, in contrast to adults, gastric lipases play a significant role in infants because of their high postprandial gastric pH (156).

It is hypothesized that, the smaller the fat globule size, the higher will be the fat digestibility, because the higher surface area of smaller fat globules will help in rapid digestion via gastrointestinal lipases (13, 18, 19, 157). Meena et al. (158) investigated the digestion of milk fat by pancreatic lipase in standardized raw cattle, buffalo, camel, and goat whole milk. The authors found that the amount of free fatty acids released followed the order: goat ~ camel > cattle > buffalo. The higher digestibility of goat and camel whole milk was believed to be due to the small size of their fat globules, as the fat globule sizes of the different milks were in the order: buffalo (3.9–7.7 μm) > cattle (1.6–4.9 μm) > goat (1.1–3.9 μm) ~ camel (1.1–2.1 μm). In addition to the fat globule size, the outer surface of the fat globule and its structure (i.e., the fat globule interface) have a crucial role to play in the digestion of fats. For example, the presence of adsorbed proteins (caused by processing such as heating and homogenization) at the interface of fat globules may result in providing easy access of lipases to the TAG core of the fat globules and thus in influencing the digestion of milk fat (157).

Some studies have also shown the influence of differences in the milk fat composition among different milks on their digestibility. For instance, Alférez et al. (159) studied the fat digestibility and metabolism in feces samples of male albino rats that were fed diets containing lyophilized goat and cattle whole milk. They found that, compared with the rats on the cattle-milk-based diet, the digestive utilization of fat was higher, and the levels of cholesterol were lower, in the rats on the goat-milk-based diet. The authors believed that the differences may have been due to the greater amounts of medium chain TAGs and the smaller fat globule sizes of the goat milk fat compared with the cattle milk fat used in their study. Similarly, Teng et al. (160) studied the *in vitro* gastric digestion of raw (non-homogenized) and homogenized cattle and sheep milk, and reported that the TAGs from both raw and homogenized sheep milk were digested by rabbit gastric lipases more rapidly than those from cattle milk; this was due to the presence of higher levels of medium chain fatty acids at the sn-1 or sn-3 position of the TAG structure in sheep milk compared with cattle milk, emphasizing that the structural characteristics of TAGs have an important role to play in their gastric digestion.

Overall, the digestibilities of the protein and fat in milk are likely to be functions of the unique compositions, protein profiles, fat compositions, casein micelle and fat globule structures, interfacial properties, mineral distributions, and physicochemical properties, all of which are likely to be affected to different degrees by the processing conditions, depending on the animal species. Although there are very few studies on the impact of the processing conditions and the milk composition of non-cattle milks in the literature, the principles of cattle milk protein coagulation and its impact on fat digestion are expected to also be applicable to non-cattle milks. However, as cattle and non-cattle milks vary in protein composition (proportion of different proteins) as well as protein-to-fat ratio, it is likely that there will be differences in the structure and consistency of the protein curd (or clot) formed from different milks, which may lead to further differences in the release of fat globules from the clot matrix of different milks. It should also be noted that the gastric and intestinal digestion conditions of infants (as well as the elderly) are different from those of adults in terms of acid secretions and enzyme (proteases and lipases) activities (155, 156, 161). Thus, relevant dynamic *in vitro* models need to be used to study the digestion of milks in different age groups, and *in vitro* results need to be ultimately corroborated based on *in vivo* observations.

## Conclusions and Recommendations for Future Research

As non-cattle milk and milk products are highly regarded as a potential source of human nutrition, they can be utilized to develop specialized dairy products for people in all age groups. Non-cattle milks are of great interest to people as well as industries, because of their perceived better nutritional properties compared with cattle milk. However, most of these presumptions are based on anecdotal reports and only little scientific research has been conducted to understand the nutritional and physicochemical properties of non-cattle milks. One widely perceived notion is the formation of soft curds in the human stomach for some non-cattle milks (such as goat, camel, horse, and donkey milk). Because of this, these milks are considered to be better digested and tolerated by people of different age groups. However, to date, no direct scientific studies have been reported and there is a knowledge gap. As cattle and non-cattle milks vary in composition and structure of the casein micelles and fat globules, they are likely to behave differently in the gastrointestinal tract, possibly affecting the kinetics of digestion and the bioavailability of nutrients. Because of differences in milk composition and the structure of the casein micelles (or fat globules), there may be differences in the curds formed by the milk of each species in the stomach, which may further affect the delivery rates of macronutrients further down the gastrointestinal tract. Furthermore, different commercial processing conditions such as pasteurization or UHT (or other heat treatments) may influence the digestion behaviors of non-cattle milks differently. Thus, in-depth scientific studies need to be conducted to understand the impact of compositional as well as structural differences in milk from different species (in their natural form as well as processed forms) on their dynamic digestion behaviors, especially focusing on their differences in curd formation as well as their disintegration properties in the stomach. Such studies will often involve *in vitro* digestion models, which where possible should be dynamic and sophisticated enough to at least include the effects of key variables known to influence food digestion. Further, the physiological relevance of such phenomena needs to be investigated in animal and human studies focusing on different age groups or people in need of targeted personalized nutrition (such as infants, the elderly, athletes or malnourished people).

## Author Contributions

DR prepared the original draft and edited the manuscript. HS critiqued and edited the original draft of the manuscript. AY and PM critically reviewed the manuscript. All authors listed have made a substantial, direct, and intellectual contribution to the conception and design of the manuscript and read and approved the final manuscript for publication.

## Conflict of Interest

The authors declare that the research was conducted in the absence of any commercial or financial relationships that could be construed as a potential conflict of interest.
